# Validity evidence for two objective structured clinical examination stations to evaluate core skills of the shoulder and knee assessment

**DOI:** 10.1186/s12909-016-0850-7

**Published:** 2017-01-13

**Authors:** Michael J. Battistone, Andrea M. Barker, J. Peter Beck, Robert Z. Tashjian, Grant W. Cannon

**Affiliations:** 1 Salt Lake City Veterans Affairs Medical Center (SLC VAMC), 500 Foothill Drive, Salt Lake City, UT 84148 USA; 2 Department of Medicine, Division of Rheumatology, University of Utah, Salt Lake City, USA; 3 Department of Family and Preventive Medicine, University of Utah, Salt Lake City, USA; 4 Department of Orthopaedics, University of Utah, Salt Lake City, USA

## Abstract

**Background:**

We developed two objective structured clinical examinations (OSCEs) to educate and evaluate trainees in the evaluation and management of shoulder and knee pain. Our objective was to examine the evidence for validity of these OSCEs.

**Methods:**

A multidisciplinary team of content experts developed checklists of exam maneuvers and criteria to guide rater observations. Content was proposed by faculty, supplemented by literature review, and finalized using a Delphi process. One faculty simulated the patient, another rated examinee performance. Two faculty independently rated a portion of cases. Percent agreement was calculated and Cohen’s kappa corrected for chance agreement on binary outcomes. Examinees’ self-assessment was explored by written surveys. Responses were stratified into 3 categories and compared with similarly stratified OSCE scores using Pearson’s coefficient.

**Results:**

A multi-disciplinary cohort of 69 examinees participated. Examinees correctly identified rotator cuff and meniscal disease 88% and 89% of the time, respectively. Inter-rater agreement was moderate for the knee (87%; k = 0.61) and near perfect for the shoulder (97%; k = 0.88). No correlation between stratified self-assessment and OSCE scores were found for either shoulder (0.02) or knee (−0.07).

**Conclusions:**

Validity evidence supports the continuing use of these OSCEs in educational programs addressing the evaluation and management of shoulder and knee pain. Evidence for validity includes the systematic development of content, rigorous control of the response process, and demonstration of acceptable interrater agreement. Lack of correlation with self-assessment suggests that these OSCEs measure a construct different from learners’ self-confidence.

**Electronic supplementary material:**

The online version of this article (doi:10.1186/s12909-016-0850-7) contains supplementary material, which is available to authorized users.

## Background

The prevalence of musculoskeletal (MSK) problems is substantial, and in 2006, data from diagnostic coding showed that MSK conditions were the most common reason for patients to visit primary care clinics in the United States (US).[1–3] Nevertheless, clinical training in MSK diseases has been widely regarded as inadequate across multiple levels of medical education in the US and abroad.[4–6] Calls for innovations in response to these training needs have come in the context of an increasing awareness of the need for reflective critique and scholarly review of initiatives in medical education.[7, 8] The US Bone and Joint Initiative’s 2011 Summit on *The Value in Musculoskeletal Care* included the following recommendation in the summary of the proceedings:

“Training programs for all health care providers should improve the knowledge, skills, and attitudes of all professionals in the diagnosis and management of musculoskeletal conditions. At present, many graduates report a deficit of knowledge of musculoskeletal conditions and competence in patient evaluation and treatment, including performance of the musculoskeletal physical examination.” [7]

In response to this call, and as part of a broad initiative to enhance MSK care, we convened a multi-disciplinary group to develop two objective structured clinical examination (OSCE) stations to facilitate training and assessment in the evaluation and management of shoulder pain and knee pain in primary care. We designed these exercises to be capstone elements within MSK educational programs, developed for students, post-graduate trainees, and practicing providers.[9–12] The purpose of the OSCEs was to assess the ability to 1) perform a systematic, efficient, and thorough physical exam, 2) recognize history and exam findings suggestive of problems commonly seen in primary care (rotator cuff disease, osteoarthritis (OA), adhesive capsulitis, and biceps tendinitis in patients with shoulder pain; OA, meniscal disease, ligamentous injury, iliotibial band pain and patellofemoral syndrome in patients with knee pain), and 3) suggest an initial management plan, including the appropriate use of imaging, corticosteroid injections, and specialty referral. Our objective in this study was to examine the evidence for validity of these two OSCE experiences.

Contemporary understanding of validity has developed recently, exchanging an older framework that had considered content, criterion (including predictive), and construct validity to be distinct concepts, for a unified hypothesis in which validity is viewed as an argument to be made—using theory, data, and logic—rather than the measureable property of an instrument or assessment tool.[13, 14] In this contemporary construct, evidence used to argue validity is drawn from multiple sources: 1) content, 2) response process, 3) internal structure, 4) relations to other variables, and 5) consequences.[15, 16].

## Methods

### Content

The OSCE stations were created by a group consisting of two orthopedic surgeons (RZT, JPB), two rheumatologists (MJB, GWC), and a primary care provider with orthopedic experience (AMB). Station content—the set of elements constituting a complete examination for the shoulder and knee—was proposed by faculty, supplemented by literature review, and finalized through a Delphi process. Checklist items representing observable exam maneuvers and the criteria for guiding rater observations to assess the quality of performance of each of these items were also developed and finalized through faculty consensus. Simulated cases representing causes of shoulder pain (rotator cuff disease, OA, adhesive capsulitis, and biceps tendinitis) and knee pain (OA, meniscal disease, ligamentous injury, iliotibial band pain and patellofemoral syndrome) commonly encountered in primary care settings were created; expert clinical faculty drafted, reviewed, and revised these cases together with the checklists and rating scales, and additional faculty reviewed and critiqued the revised versions. Exacting specifications detailed all the essential clinical information to be portrayed by the simulated patient (SP).

### Response process

OSCE scores were collected in the context of intensive structured educational programs developed for trainees and practicing primary care providers.[11, 12] To promote accuracy of responses to assessment prompts, and to ensure strong data collection, one faculty member served as the SP (MJB) and another as the rater (AMB). OSCEs were conducted in clinical exam rooms, and ratings were recorded in real time. Any and all questions regarding the performance of specific exam maneuvers or the quality of the technique were resolved between the two faculty immediately following the exercise.

A scoring rubric was designed to produce five total possible points for the shoulder OSCE. The elements were distributed and organized into five domains: observation, palpation, range of motion, motor function of the rotator cuff, and provocative testing. Each domain was assigned a factor weight by clinical experts on the basis of their assessment of the importance that each domain contributed to clinical decision-making. For example, testing the rotator cuff motor function was assigned a factor of 1.5. This domain was a greater factor weight than that assigned to provocative testing—factor of 1—because if weakness of the rotator cuff is noted during the physical exam, magnetic resonance imaging (MRI) may be considered, whereas a positive Speed’s or Yergason’s test suggesting biceps tendinitis would not be expected to lead to advanced imaging. Each of the items within each domain was weighted equally. When the rater scored the OSCE, if a skill was not performed the item was scored as “0.” If the skill was attempted but the technique was not adequate, it was scored as “1;” if performed correctly, it was scored as “2.” The score within each domain was the percentage of possible points within that domain.

A similar five point scoring rubric was developed for the knee OSCE, with elements distributed and organized across five domains: observation, range of motion, palpation, stability testing, and provocative testing. As for the shoulder station, each domain is assigned a factor weight, to reflect differences in how the relative maneuvers might have greater or lesser impact on clinical decisions. Rating and scoring the knee OSCE followed the same procedure used in the shoulder station.

### Internal structure

To establish interrater agreement, two faculty members (AMB, MJB) independently rated 10% of the cases. Inter-rater agreement was calculated and Cohen’s kappa corrected for chance agreement on binary outcomes.

### Relations to other variables

Relationship to self-assessment of ability to evaluate shoulder pain and knee pain was explored with written surveys, using Likert scales anchored at five points ranging from 1 (Strongly Disagree) to 5 (Strongly Agree). Five items related to the shoulder and 5 to the knee. In addition to using these in traditional pre-course and post-course measurements, participants were asked—after the course ended—to retrospectively rate their pre-course proficiency—in effect, capturing information that trainees “didn’t know they didn’t know.” [17, 18] Responses were averaged for each of the 5 items; averaged responses were then stratified across 3 categories of self-assessed ability—low, medium, and high—and compared with similarly stratified OSCE scores.

This project was reviewed by the Institutional Review Board of the University of Utah and was determined to meet the definition of a quality improvement study but not the definition of research with human subjects, and was classified as exempt. Written consent was not requested.

## Results

### Content evidence

Final versions of the shoulder (21 items) and knee (25 items) checklists are shown in Tables 1 and 2, respectively. Table 1 Legend: Shoulder Examination Checklist. Table 2 Legend: Knee Examination Checklist. Individual items were grouped into 5 domains: “observation”, “palpation”, “range of motion”, “motor function of rotator cuff”, and “provocative testing” for the shoulder; “observation”, “range of motion”, “palpation”, “stability testing”, and “provocative testing” for the knee. Videos demonstrating the performance of the complete exam were developed (AMB, MJB) to accompany these checklists as teaching tools.[9] Copies of these videos are available as supplementary materials (see Shoulder Exam Small 2014.mov and Knee Exam Small 2014.mov) and online [19, 20].

**Table 1:** Shoulder Physical Examination

		Examination	Performed	Technique Adequate
	From Behind			
1		Observation		
		Adequate exposure	0 1 2	Observe as they disrobe for degree of discomfort
		General	0 1 2	Symmetry, scars, skin lesions, erythema, edema, atrophy
		Scapular winging	0 1 2	Patient raises arms bilaterally Wall press
2		Palpation		
		Sternoclavicular joints	0 1 2	
		Acromioclavicular joints	0 1 2	
		Biceps tendons	0 1 2	
		Subacromial space	0 1 2	Lateral and posterolateral
	Facing Patient			
3		Range of Motion		
4		Motor Function of Rotator Cuff		
		Bilateral	0 1 2	
	Supraspinatus	ROM: Active abduction in scapular plane Painful arc (>90°) Drop arm test	0 1 2	Scapular plane Neutral rotation (thumbs to ceiling) Allow for full active adduction
		Motor: Empty Can Test	0 1 2	Scapular plane Full pronation (thumbs to floor) Resisted abduction at 90° or less
	Infraspinatus	ROM: Active external rotation	0 1 2	Elbows at side
		Motor: Active external rotation against resistance	0 1 2	Elbows at side Start with hands near midline
		Unilateral		
	Subscapularis	Motor: Belly Press Test	0 1 2	Hand on abdomen Elbow anterior to midline Examiner pulls at forearm Watch for elbow to drop
		ROM: Active internal rotation along spine	0 1 2	Observe patient from behind
		Motor: Lift Off Test	0 1 2	Hand at lumbar spine Actively lifts arm off back against resistance at wrist
	Teres Minor	ROM: Active external rotation with 90° shoulder abduction and 90° elbow flexion	0 1 2	90° shoulder abduction 90° elbow flexion Active external rotation
		Motor: Hornblower’s Test	0 1 2	External rotation as above against resistance
5		Provocative Testing		
		Impingement Testing		
		Hawkin’s Test	0 1 2	Shoulder 90° abduction Scapular plane 90° elbow flexion Internal rotation + horizontal adduction
		Neer’s Test	0 1 2	Elbow extended Full pronation Maximal passive forward elevation of shoulder with scapular stabilization
		Biceps Testing		
		Speed’s Test	0 1 2	60° forward elevation Hand in supination 20–30° elbow flexion Apply downward pressure to forearm
		Yergason’s Test	0 1 2	Elbow at side, 90° flexion Palm in supination Resisted supination
		AC Joint Testing		
		Cross-arm Test	0 1 2	Active horizontal adduction

*Note*: check passive range of motion if active is limited. This will identify mechanical block of motion versus shoulder weakness. *ROM* range of motion, *AC* acromioclavicular

**Table 2:** Knee Physical Examination

	Examination	Performed	Technique Adequate
1	Observation		
	Standing	0 1 2	Gait, alignment, popliteal fossa
	Supine position, knee adequately exposed	0 1 2	Alignment, atrophy, lesions, scars, erythema
	Effusion	0 1 2	Full extension, medial/lateral gutters
2	Range of Motion		
	Extension/Flexion 0–140°	0 1 2	
	Hip IR (30°) and ER (60°)	0 1 2	
3	Palpation		
	Flex to 90° with heel resting on table	0 1 2	
	Quadriceps tendon	0 1 2	
	Patellar tendon	0 1 2	
	Tibial tubercle	0 1 2	
	Lateral joint line	0 1 2	
	Lateral femoral epicondyle	0 1 2	(proximal LCL and ITB)
	Fibular head	0 1 2	(distal LCL)
	Medial joint line	0 1 2	
	Medial femoral epicondyle	0 1 2	(proximal MCL)
	Medial tibia	0 1 2	(distal MCL)
	Pes anserine bursa	0 1 2	
4	Stability Testing		
	Posterior Cruciate Ligament Posterior Drawer	0 1 2	Knee flexed to 90° Examiner stabilizes foot Thumbs on anterior tibia, translate posterior
	Anterior Cruciate Ligament Anterior drawer	0 1 2	Knee flexed to 90° Examiner stabilizes foot Thumbs on anterior tibia, translate anterior
	Anterior Cruciate Ligament Lachman Test	0 1 2	Knee flexed to 30° Hands near joint line Anterior tibial translation
	Medial Collateral Ligament (Valgus stress)	0 1 2	30° flexion
	Lateral Collateral Ligament (Varus stress)	0 1 2	30° flexion
5	Provocative Testing		
	Meniscus Testing		
	McMurray Test Medial Meniscus	0 1 2	Fingers on posteromedial joint line Full knee flexion External rotation sweep and slow leg extension
	McMurray Test Lateral Meniscus	0 1 2	Fingers on posterolateral joint line Full knee flexion Internal rotation sweep and slow leg extension
	Patellofemoral Assessment (knee in extension)		
	Palpation of medial and lateral patellar facets	0 1 2	
	Patellar Compression Test	0 1 2	Active quadriceps contraction Repeat with posterior patellar compression
	IT Band Assessment		
	Noble Compression Test	0 1 2	Palpate lateral femoral epicondyle Passive knee ROM (pain at 30°)

*IR* internal rotation, *ER* external rotation, *LCL* lateral collateral ligament, *MCL* medial collateral ligament, *ITB* iliotibial band


Additional file 1: Shoulder Exam Small 2014.
Additional file 2: Knee Exam Small 2014.


### Response process evidence

A multi-disciplinary cohort of 69 trainees participated in the OSCEs in 2014–15 Table 3.

**Table 3:** Students and trainees participating in the OSCE

	N
Post-graduates	
Internal Medicine (PGY-1)	34
Physical Med & Rehab (PGY-3)	3
Orthopedics (PGY-1)	2
Occupational Medicine (PGY-2)	5
Physical Therapy Residents	2
Students	
Physician Assistant	11
Advance Practice Nursing	5
Medicine (MS4)	7
TOTAL	69

Using the examination approach in the checklists, 88% of the trainees correctly identified rotator cuff pathology and 89% of them correctly diagnosed meniscal disease.

### Internal structure evidence

Observed inter-rater agreement was 87% for items on the knee checklist, and 97% for those on the shoulder Table 4.

**Table 4:** Inter-rater agreement for shoulder and knee checklist items

Shoulder		Rater 1		
		Performed	Not Performed	Total
Rater 2	Performed	118	1	119
	Not Performed	4	24	28
	Total	122	25	147
Knee		Rater 1		
		Performed	Not Performed	Total
Rater 2	Performed	126	14	140
	Not Performed	9	26	35
	Total	171	30	175

Observed Agreement = (118 + 24)/147 = 0.97

Chance Agreement = 0.70

Cohen’s kappa = 0.9 (“Almost perfect”)

Observed Agreement = (126 + 26)/175 = 0.87

Chance Agreement = 0.66

Cohen’s kappa = 0.6 (“Moderate”)

Kappa coefficients indicated moderate agreement for the knee (0.6) and near perfect agreement for the shoulder (0.9), according to a commonly cited scale [21].

### Relations to other variables evidence

Sixty nine pre-course, 67 post-course, and 63 retrospective pre-course surveys were collected (response rates of 100, 91 and 97%, respectively); mean self-assessment ratings are shown in Table 5.

**Table 5:** Pre-course and post-course (including retrospective pre-course) self-assessment ratings

	Mean Pre-course Ratings ± s.dev		Mean Post-course Ratings (*n* = 67)
	Prospective (*n* = 69)	Retrospective (*n* = 63)	
Shoulder Pain			
*I can examine and diagnose shoulder pain without MRI*	2.8 ± 1.0	2.3 ± 1.0	4.6 ± 0.6
*I can evaluate patients effectively*	2.8 ± 0.9	2.4 ± 1.0	4.6 ± 0.6
*I can develop an appropriate plan*	2.9 ± 0.9	2.2 ± 0.9	4.3 ± 0.7
*I understand when to order imaging*	3.0 ± 0.8	2.4 ± 1.0	4.4 ± 0.6
*I understand when to refer*	3.0 ± 0.9	2.4 ± 1.0	4.5 ± 0.6
Knee Pain			
*I can examine and diagnose knee pain without MRI*	3.3 ± 0.9	2.7 ± 0.9	4.4 ± 0.6
*I can evaluate patients effectively*	3.2 ± 0.9	2.6 ± 0.9	4.5 ± 0.6
*I can develop an appropriate plan*	3.1 ± 0.8	2.6 ± 1.0	4.3 ± 0.6
*I understand when to order imaging*	3.4 ± 0.9	2.6 ± 1.0	4.5 ± 0.6
*I understand when to refer*	3.3 ± 0.9	2.6 ± 0.9	4.5 ± 0.5

Relationship of stratified self-assessment and OSCE scores is shown in Table 6.

**Table 6:** Relationship of stratified OSCE scores and self-assessment ratings

Shoulder		OSCE Rating			Total
		Low (n)	Med (n)	High (n)	
Self-Assessment Rating	Low (n)	0	0	5	5
	Med (n)	4	5	20	29
	High (n)	5	5	25	35
		9	10	50	69
Knee		OSCE Rating			
		Low	Med	High	
Self-Assessment Rating	Low	0	0	2	2
	Med	11	12	12	35
	High	10	13	9	32
		21	25	23	69

Pearson’s coefficient indicated no correlation for either the shoulder (0.02) or the knee (−0.07)

## Discussion

We have developed a systematic, efficient, and feasible method of organizing, teaching, and evaluating the physical examination of the shoulder and the knee. This paper presents validity evidence supporting the use of these examination checklists and OSCE stations in the context of an educational program focused on strengthening these clinical skills.

Several recent reports have been published, which describe the development and use of OSCE stations and checklists in the context of MSK and rheumatology; the two most recent of these emphasize the importance of developing consensus among educators regarding the elements of these important teaching and assessment tools.[22–28] There are many possible techniques used in examining the MSK system, and a recent review by Moen et al. reported that at least 109 specific maneuvers for the shoulder have been described.[29] Although some individual studies have reported sensitivity and specificity properties for these maneuvers that may seem reasonable, other studies have arrived at different results, and a recent Cochrane review has not found sufficient evidence to recommend any examination element, likely due to “extreme diversity” in techniques compared to the original descriptions.[30] Many studies have not examined combinations of individual elements into a systematic, synthetic approach; some even question the relevance and role of the physical examination altogether, in contrast with the summary recommendation of the US Bone and Joint Initiative.[31, 32] No group has yet proposed a detailed checklist of elements for the physical exam of the shoulder for use in a multidisciplinary educational program.

Our study has several strengths. First, the content of our instruments was developed using a well-defined process, grounded in an explicit theoretical and conceptual basis—that in order to be effective the physical exam must balance thoroughness with feasibility. Our checklists represent those elements that were identified in the literature and finalized in a systematic item review by a multidisciplinary panel of experts representing orthopedics, rheumatology, and primary care. Second, the strength of our methods to control the response process and preserve a coherent internal structure within these OSCEs is demonstrated by the high rate of accuracy in identifying simulated rotator cuff and meniscal pathology, as well as good interrater agreement of faculty assessors. Finally, we have addressed the relationship of these structured observations of clinical skill to written self-assessments.

Further development of this educational initiative will involve exploring the use of these tools in several additional settings: 1) a national continuing professional education initiative to strengthen the evaluation and management of MSK conditions in primary care, 2) a dedicated simulation facility, 3) a national initiative for a rheumatology OSCE, and 4) individual institutions providing undergraduate and graduate medical education experiences.[10, 12, 24] This exploration will involve work to examine validity evidence informing the interpretation of scores in each of these contexts.

We acknowledge several limitations to our study. First, we have not examined the relationship of these OSCEs to other assessments of knowledge, including written examinations. We are currently developing additional methods of evaluation, including multiple choice questions that will evaluate content knowledge. Second, we do not currently have evidence of consequence to inform our validity hypothesis. Sources of consequence evidence might include more appropriate use of high-cost imaging, better prioritization of referrals to physical therapy, surgery, or specialty care, and more precise documentation of the physical exam. Finally, our study examines evidence of the performance of these assessments within a single institution. It is believed that these teaching and assessment tools are generalizable, and offer a valuable resource at additional sites.

## Conclusions

In summary, we have presented evidence of validity supporting the use of these shoulder and knee OSCEs as a capstone element of a structured educational program designed to strengthen the evaluation and management of common MSK complaints. This initial critical review of these assessment tools prepares the way for dissemination of these OSCEs to other institutions, learning platforms, and contexts, where additional examination of the experiences of implementation will be important to determine generalizability and feasibility.

## Abbreviations

MRI: Magnetic resonance imaging
MSK: Musculoskeletal
OA: Osteoarthritis
OSCE: Objective structured clinical examination
SP: Simulated patient
US: United States
VA: Veterans Affairs

## References

[1] Helmick CG, Felson DT, Lawrence RC, Gabriel S, Hirsch R, Kwoh CK, Liang MH, Kremers HM, Mayes MD, Merkel PA (2008). Estimates of the prevalence of arthritis and other rheumatic conditions in the United States.Part I. Arthritis Rheum.

[2] Lawrence RC, Felson DT, Helmick CG, Arnold LM, Choi H, Deyo RA, Gabriel S, Hirsch R, Hochberg MC, Hunder GG (2008). Estimates of the prevalence of arthritis and other rheumatic conditions in the United States. Part II. Arthritis Rheum.

[3] Cherry DK, Hing E, Woodwell DA, Rechtsteiner EA. National Ambulatory Medical Care Survey: 2006 summary. Natl Health Stat Report. 2008;(3):1–39. 18972720

[4] Glazier RH, Dalby DM, Badley EM, Hawker GA, Bell MJ, Buchbinder R, Lineker SC (1996). Management of the early and late presentations of rheumatoid arthritis: a survey of Ontario primary care physicians. CMAJ.

[5] Dequeker J, Rasker JJ, Woolf AD (2000). Educational issues in rheumatology. Baillieres Best Pract Res Clin Rheumatol.

[6] Monrad SU, Zeller JL, Craig CL, Diponio LA (2011). Musculoskeletal education in US medical schools: lessons from the past and suggestions for the future. Curr Rev Musculoskelet Med.

[7] Gnatz SM, Pisetsky DS, Andersson GB (2012). The value in musculoskeletal care: summary and recommendations. Semin Arthritis Rheum.

[8] Regehr G (2010). It’s NOT rocket science: rethinking our metaphors for research in health professions education. Med Educ.

[9] Battistone MJ, Barker AM, Grotzke MP, Beck JP, Berdan JT, Butler JM, Milne CK, Huhtala T, Cannon GW (2016). Effectiveness of an interprofessional and multidisciplinary musculoskeletal training program. J Grad Med Educ.

[10] Battistone MJ, Barker AM, Grotzke MP, Beck JP, Lawrence P, Cannon GW (2016). “Mini-residency” in musculoskeletal care: a national continuing professional development program for primary care providers. J Gen Intern Med.

[11] Battistone MJ, Barker AM, Lawrence P, Grotzke MP, Cannon GW (2016). Mini-residency in musculoskeletal care: an interprofessional, mixed-methods educational initiative for primary care providers. Arthritis Care Res (Hoboken).

[12] Battistone MJ, Barker AM, Okuda Y, Gaught W, Maida G, Cannon GW. Teaching the Teachers: Report of an Effective Mixed-Methods Course Training Clinical Educators to Provide Instruction in Musculoskeletal Care to Other Providers and Learners in Primary Care [abstract]. Arthritis Rheumatol. 2015;67(suppl 10).

[13] Downing SM (2003). Validity: on meaningful interpretation of assessment data. Med Educ.

[14] Cook DA, Beckman TJ (2006). Current concepts in validity and reliability for psychometric instruments: theory and application. Am J Med.

[15] Messick S, Linn RL (1989). Educational Measurement. New York: American Council on Education.

[16] American Educational Research Association, American Psychological Association, National Council on Measurement in Education, Joint Committee on Standards for Educational and Psychological Testing (U.S.) (1999). Standards for educational and psychological testing.

[17] Eva KW, Regehr G (2005). Self-assessment in the health professions: a reformulation and research agenda. Acad Med.

[18] Nagler M, Feller S, Beyeler C (2012). Retrospective adjustment of self-assessed medical competencies - noteworthy in the evaluation of postgraduate practical training courses. GMS Z Med Ausbild.

[19] Shoulder Physical Exam [https://www.youtube.com/watch?v=dzHFwkXBmvU]

[20] Knee Physical Exam for Primary Care Providers [https://www.youtube.com/watch?v=YhYrMisCrWA]

[21] Landis JR, Koch GG (1977). The measurement of observer agreement for categorical data. Biometrics.

[22] Berman JR, Lazaro D, Fields T, Bass AR, Weinstein E, Putterman C, Dwyer E, Krasnokutsky S, Paget SA, Pillinger MH (2009). The New York city rheumatology objective structured clinical examination: five-year data demonstrates its validity, usefulness as a unique rating tool, objectivity, and sensitivity to change. Arthritis Rheum.

[23] Branch VK, Graves G, Hanczyc M, Lipsky PE (1999). The utility of trained arthritis patient educators in the evaluation and improvement of musculoskeletal examination skills of physicians in training. Arthritis Care Res.

[24] Criscione-Schreiber LG, Sloane RJ, Hawley J, Jonas BL, O’Rourke KS, Bolster MB (2015). Expert panel consensus on assessment checklists for a rheumatology objective structured clinical examination. Arthritis Care Res (Hoboken).

[25] Hassell AB, West Midlands Rheumatology S, Training C (2002). Assessment of specialist registrars in rheumatology: experience of an objective structured clinical examination (OSCE). Rheumatology (Oxford).

[26] Raj N, Badcock LJ, Brown GA, Deighton CM, O’Reilly SC (2006). Undergraduate musculoskeletal examination teaching by trained patient educators--a comparison with doctor-led teaching. Rheumatology (Oxford).

[27] Ryan S, Stevenson K, Hassell AB (2007). Assessment of clinical nurse specialists in rheumatology using an OSCE. Musculoskeletal Care.

[28] Vivekananda-Schmidt P, Lewis M, Hassell AB, Coady D, Walker D, Kay L, McLean MJ, Haq I, Rahman A (2007). Validation of MSAT: an instrument to measure medical students’ self-assessed confidence in musculoskeletal examination skills. Med Educ.

[29] Moen MH, de Vos RJ, Ellenbecker TS, Weir A (2010). Clinical tests in shoulder examination: how to perform them. Br J Sports Med.

[30] Hanchard NC, Lenza M, Handoll HH, Takwoingi Y (2013). Physical tests for shoulder impingements and local lesions of bursa, tendon or labrum that may accompany impingement. Cochrane Database Syst Rev.

[31] Hegedus EJ, Goode A, Campbell S, Morin A, Tamaddoni M, Moorman CT, Cook C (2008). Physical examination tests of the shoulder: a systematic review with meta-analysis of individual tests. Br J Sports Med.

[32] May S, Chance-Larsen K, Littlewood C, Lomas D, Saad M (2010). Reliability of physical examination tests used in the assessment of patients with shoulder problems: a systematic review. Physiotherapy.

